# Idiopathic Retinitis, Vasculitis, Aneurysms, and Neuroretinitis (IRVAN): Early Treatment Saves Sight

**DOI:** 10.7759/cureus.23049

**Published:** 2022-03-10

**Authors:** Aini Mohd Azmi, Wan Haslina Wan Abdul Halim, Maizan Yaakub, Rosiah Muda

**Affiliations:** 1 Ophthalmology, Hospital Canselor Tuanku Muhriz UKM (Universiti Kebangsaan Malaysia), Kuala Lumpur, MYS; 2 Ophthalmology, Hospital Sultanah Nur Zahirah, Kuala Terengganu, MYS

**Keywords:** oral prednisolone, retinal photocoagulation, macula edema, irvan, occlusive vasculitis, arteriolar aneurysm, neuroretinitis

## Abstract

We report a rare case of bilateral Idiopathic Retinitis, Vasculitis, Aneurysms, and Neuroretinitis (IRVAN) with occlusive vasculitis.

A 28-year-old female presented with sudden decreased vision in her left eye for three days. Visual acuity in the right eye was 6/6, whereas it was 6/9 in the left eye. The anterior segment was examined and found to be normal. A fundus examination of the right eye showed an arteriolar aneurysm on the optic disc, vascular sheathing, and generalized retinal pigment epithelial atrophy. The left eye was in worse condition, with a swollen optic disc, disc hemorrhage, multiple arteriolar aneurysms, hard exudates at the peripapillary and macular region, peripheral vasculitis, neovascularization, and vitreous hemorrhage. Optical coherence tomography revealed mild cystoid macula edema (CME) in both eyes. Fluorescein angiography of both eyes demonstrated arteriolar aneurysms, vascular leakage, and peripheral ischemia. There was additional leakage from new vessels and masking secondary to vitreous hemorrhage in the left eye. The results of the systemic evaluation and extensive laboratory testing were negative. She had bilateral retinal photocoagulation and was administered oral prednisolone later with slow tapering due to increasing CME. Her eye condition did not worsen, and she maintained good vision in both eyes.

IRVAN, even though rare, should be suspected in patients with occlusive vasculitis, arteriolar aneurysm, and macula exudation. Since the nature of the disease is more aggressive than other ischemic retinopathies, early detection, intervention, and close follow-up are crucial to prevent rapid visual loss.

## Introduction

Idiopathic Retinitis, Vasculitis, Aneurysms, and Neuroretinitis (IRVAN) is a rare but well-recognized clinical entity. It typically affects young, healthy individuals, has a female predominance, and is not associated with systemic abnormalities [1]. The acronym IRVAN highlights the most prominent clinical features of this syndrome [1]. If left untreated, it may lead to severe bilateral visual loss [2-3].

This case highlights the importance of early treatment initiation to preserve good vision in patients with IRVAN.

This case report was presented as a poster at the 10th MSO Annual Scientific Meeting in conjunction with 34th Malaysia-Singapore Joint Ophthalmic Congress 2019, Malaysia, March 22-24, 2019.

## Case presentation

**Figure 1:**
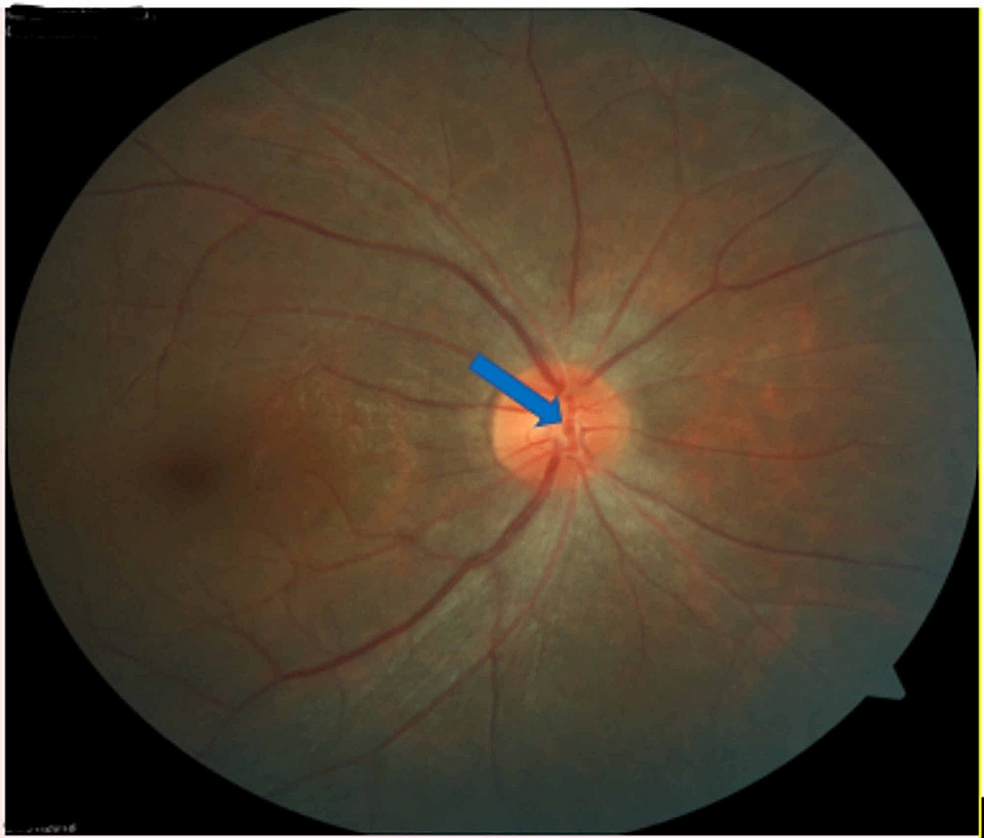
Right eye arteriolar aneurysm on the disc

**Figure 2:**
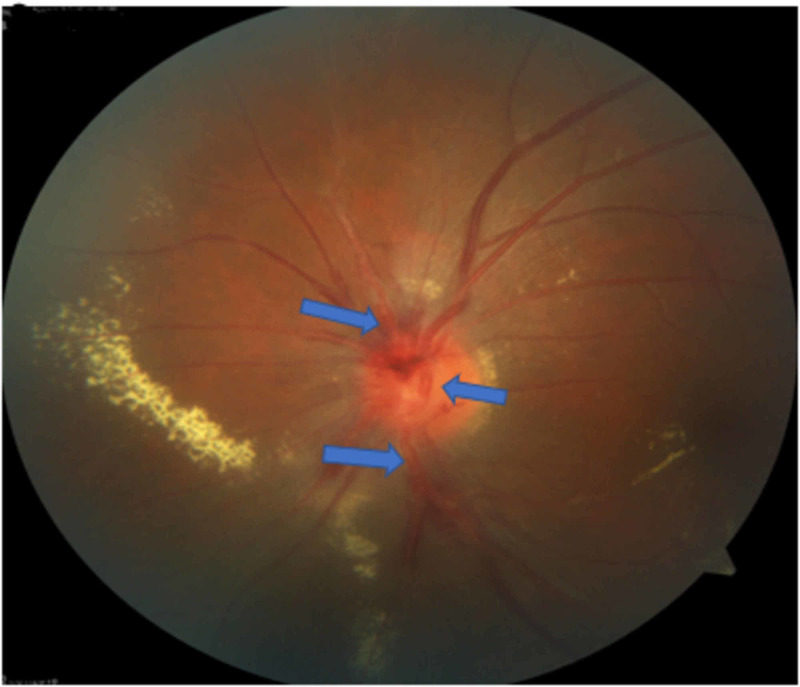
Left eye multiple arteriolar aneurysms with hard exudates at the peripapillary and macula region

A 28-year-old female presented with sudden decreased vision in her left eye for three days. Visual acuity in the right eye was 6/6, whereas it was 6/9 in the left eye. The anterior segment was examined and found to be normal. A fundus examination of the right eye showed arteriolar aneurysm on the disc (Figure 1), vascular sheathing, and generalized retinal pigment epithelial atrophy. The left eye was in worse condition with a swollen optic disc, disc hemorrhage, multiple arteriolar aneurysms, hard exudates at the peripapillary and macular regions (Figure 2), peripheral vasculitis, neovascularization, and vitreous hemorrhage.

**Figure 3:**
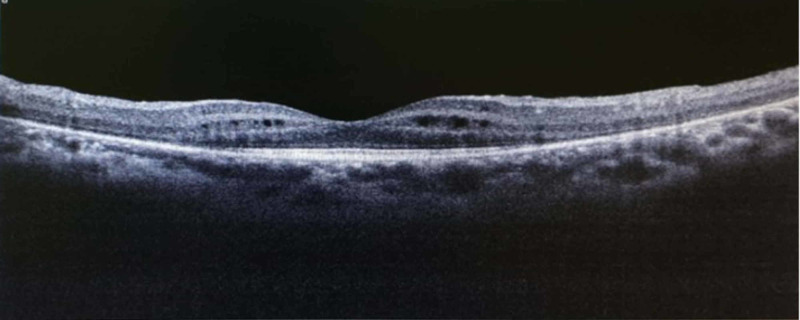
Right cystoid macula edema

**Figure 4:**
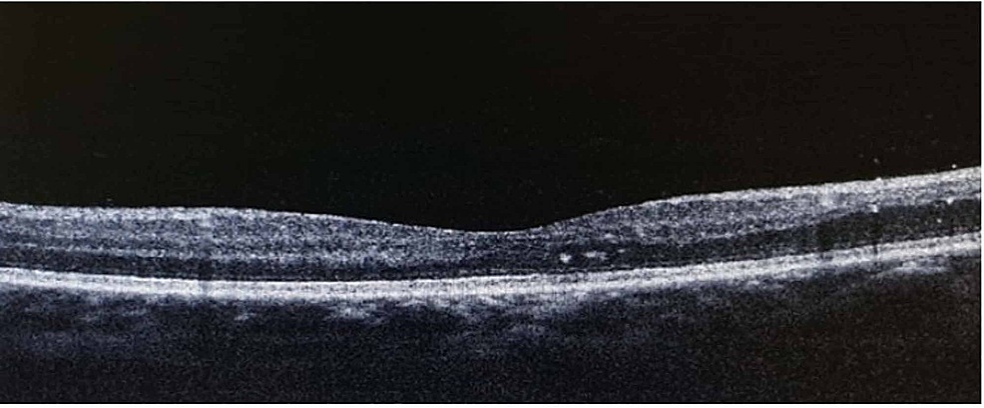
Left cystoid macula edema

**Figure 5:**
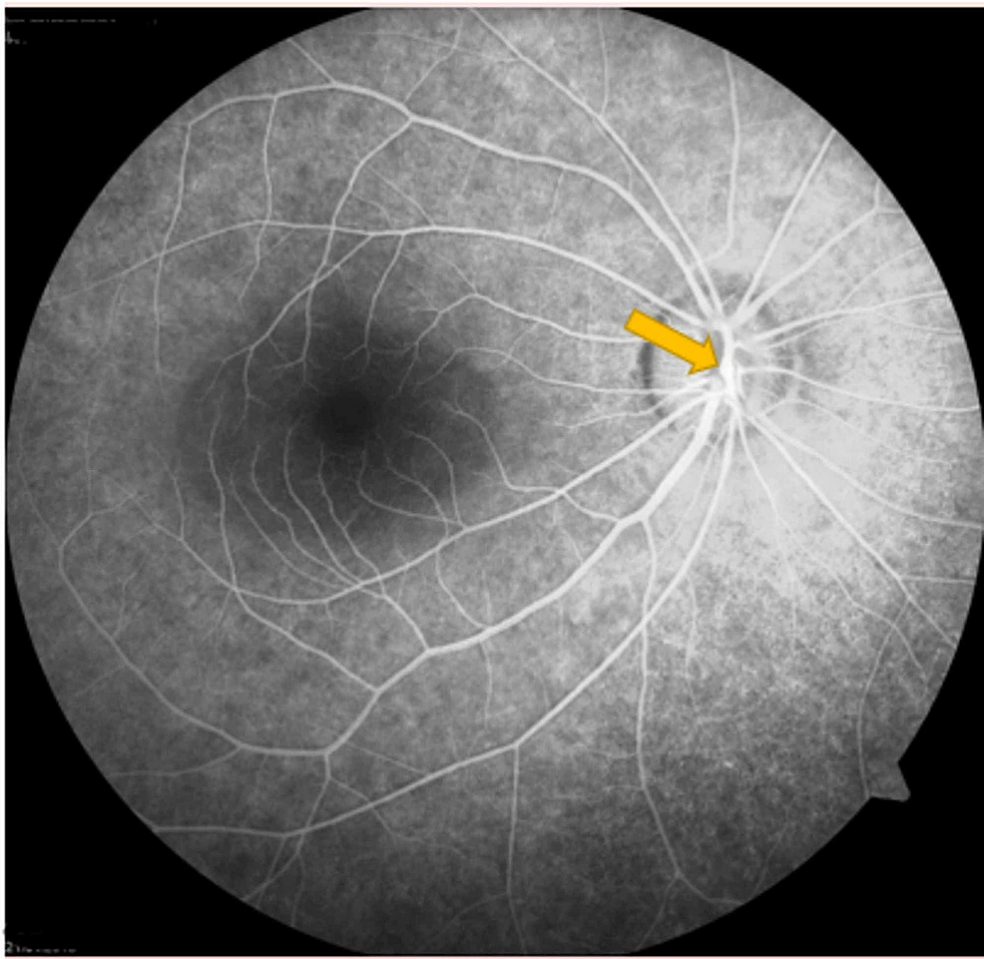
Venous phase – right eye arteriolar aneurysms and vascular leakage

**Figure 6:**
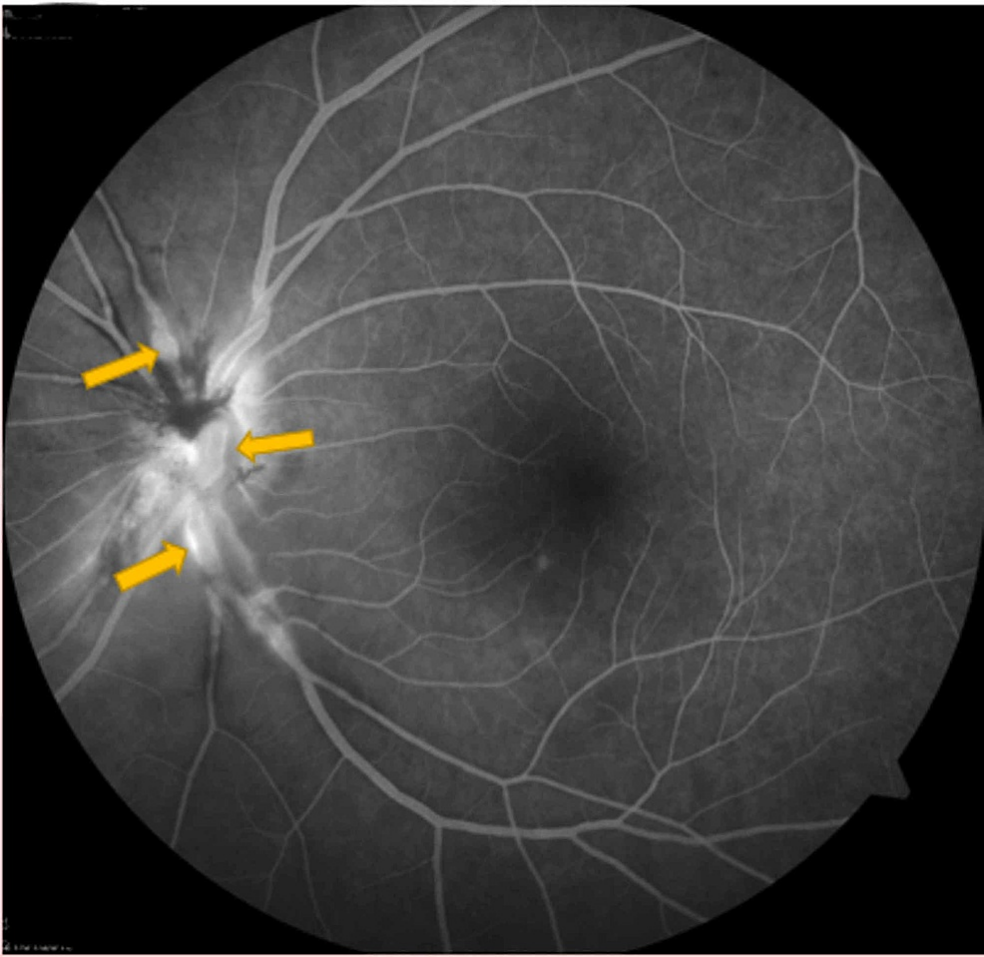
Venous phase – left eye arteriolar aneurysms and vascular leakage

**Figure 7:**
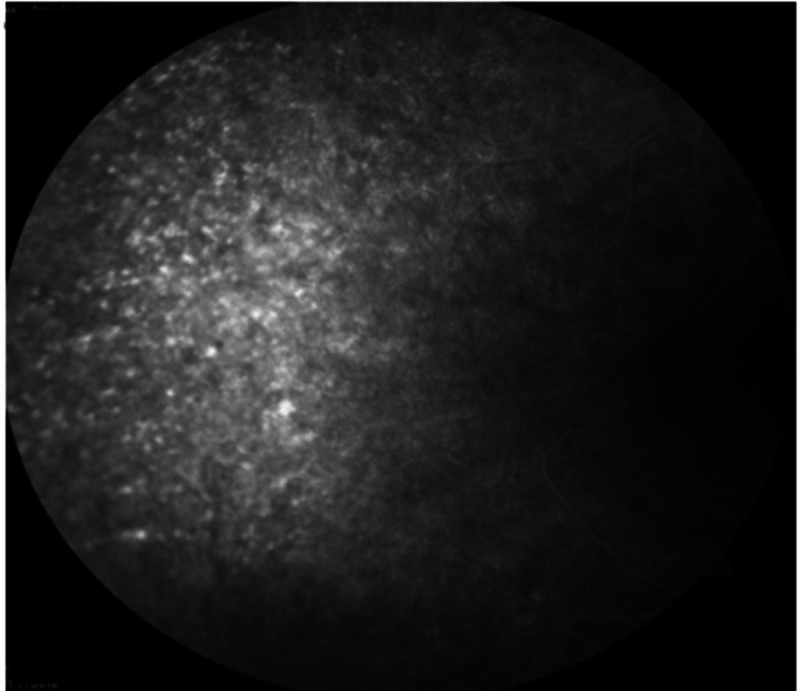
Venous phase — right eye peripheral capillary fallout with generalized window defect secondary to retinal pigment epithelial atrophy

**Figure 8:**
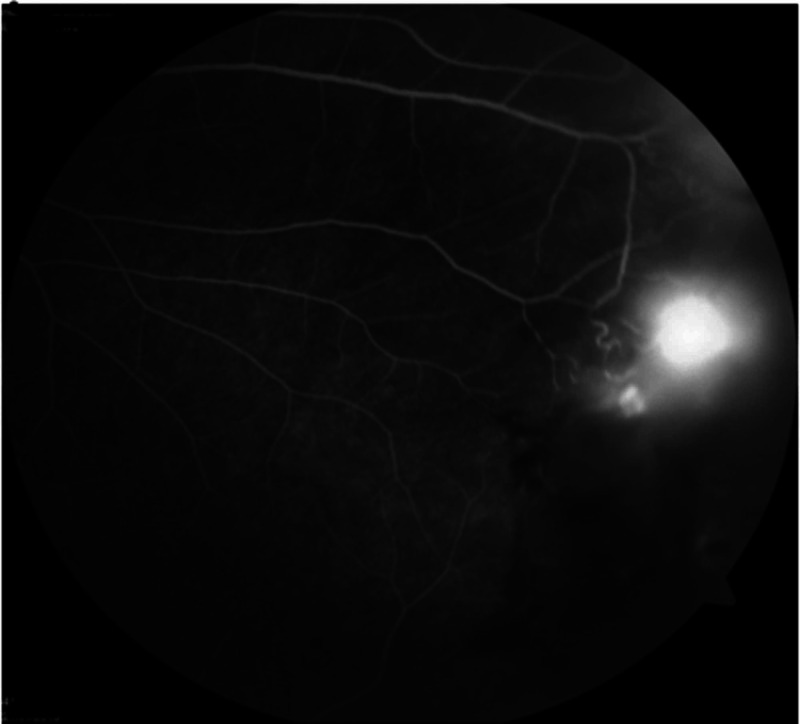
Venous phase – left eye new vessels leakage, masking secondary to vitreous hemorrhage, peripheral capillary fallout

Optical coherence tomography revealed the presence of mild cystoid macula edema (CME) (Figures 3-4) and fundus fluorescein angiography demonstrated arteriolar aneurysms, vascular leakage (Figures 5-6), and peripheral ischemia in both eyes (Figures 7-8). Leakage from new vessels and masking secondary to vitreous hemorrhage was observed in the left eye (Figure 8). Systemic evaluation and extensive laboratory testing were negative. This included full blood count, erythrocyte sedimentation rate, liver function test, renal profile, anti-nuclear antibody, rheumatoid factor, syphilis and leptospirosis serology, anti-neutrophil cytoplasmic antibody (P-ANCA and C-ANCA), complement C3/C4, chest X-ray, urine full examination microscopy examination (FEME), and Mantoux test.

The patient immediately underwent bilateral retinal photocoagulation. Due to increasing CME, high-dose oral prednisolone was administered with an initial dose of 40 mg OD and subsequently tapered down over the course of 11 months. Our patient did not demonstrate any worsening of her eye condition in subsequent follow-ups and maintained good vision in both eyes.

## Discussion

The acronym IRVAN (Idiopathic Retinitis, Vasculitis, Aneurysm, Neuroretinitis) refers to the most prominent clinical feature of this syndrome [1]. IRVAN is diagnosed using three major criteria (retinal vasculitis, aneurysmal dilations at arterial bifurcations, and neuroretinitis) and three minor criteria (peripheral capillary nonperfusion, retinal neovascularization, and macular exudation) [3], all of which are present in this patient. This unique constellation of findings that characterize IRVAN has resulted in increased recognition of this syndrome among retinal experts [3]. Early detection is vital since IRVAN can quickly develop to visual loss due to ischemic sequelae or massive exudation [4].

The time to initiate treatment of IRVAN is controversial, as there have been no large randomized prospective clinical trials done for IRVAN. However, some researchers recommended initiating panretinal laser photocoagulation before or shortly after the onset of any neovascularization [3]. Another study found that because the nature of the disease is more aggressive than other ischemic retinopathies, early intervention in the form of prompt laser treatment without waiting for neovascularization to develop is critical in these patients [4]. As a result, our patient underwent early retinal photocoagulation for both eyes. Laser photocoagulation has been shown to reduce the risk of visual loss in patients with ischemia and neovascularization. However, if treatment was initiated during more advanced stages of the disease, the patient may still lose further vision or develop neovascular glaucoma [3].

The role of steroids in IRVAN remains uncertain at this time [4]. However, a study done by Empeslidis et al. reported a marked reduction in macular thickness with steroid implant and immunosuppressive medication [5]. In this patient, macula edema was resolved after taking an oral corticosteroid. Other than administering steroids orally, current advancement in treatment also advocates the usage of a dexamethasone implant. Recent studies have shown that an intravitreal slow-release dexamethasone implant can help eyes with IRVAN syndrome improve their visual performance and macular structure [3,6]. The majority of the eyes that were reported had already undergone several treatments, such as retinal laser photocoagulation, anti-vascular endothelial growth factor (VEGF) injections, and pars plana vitrectomy [3,6]. In most eyes, a dexamethasone implant resulted in a slight increase in visual acuity but a significant reduction in macular exudation [3,6].

## Conclusions

This case has indeed proven that a directed and appropriate physical examination with a high index of suspicion, plays an important role in identifying an uncommon ophthalmologic condition, which, in this case, led to timely intervention. IRVAN, even though rare, should be suspected in patients with occlusive vasculitis, arteriolar aneurysm, and macula exudation. Since the nature of the disease is more aggressive than other ischemic retinopathies, early detection, intervention, and close follow-up are crucial to prevent rapid visual loss. Locally, this case is among the ones that had received the earliest treatment and hence had a good visual outcome.

## References

[1] Witkin AJ, Hahn P, Murray TG (2020). Occlusive retinal vasculitis following intravitreal brolucizumab. J Vitreoretin Dis.

[2] Wang P, Chin EK, Almeida DR (2021). Idiopathic retinal arterial occlusive vasculitis in the setting of multiple arterial occlusions. Am J Ophthalmol Case Rep.

[3] Ali Khan H, Ali Khan Q, Shahzad MA (2022). Comprehensive overview of IRVAN syndrome: a structured review of case reports and case series. Ther Adv Ophthalmol.

[4] Bajgai P, Katoch D, Dogra MR, Singh R (2017). Idiopathic retinal vasculitis, aneurysms, and neuroretinitis (IRVAN) syndrome: clinical perspectives. Clin Ophthalmol.

[5] Empeslidis T, Banerjee S, Vardarinos A, Konstas AG (2013). Dexamethasone intravitreal implant for idiopathic retinal vasculitis, aneurysms, and neuroretinitis. Eur J Ophthalmol.

[6] Saatci AO, Ayhan Z, Takeş Ö, Yaman A, Bajin FM (2015). Single bilateral dexamethasone implant in addition to panretinal photocoagulation and oral azathioprine treatment in IRVAN syndrome. Case Rep Ophthalmol.

